# Circular RNA-regulated autophagy is involved in cancer progression

**DOI:** 10.3389/fcell.2022.961983

**Published:** 2022-09-14

**Authors:** Xuejian Zhou, Juntao Lin, Feifan Wang, Xianwu Chen, Yan Zhang, Zhenghui Hu, Xiaodong Jin

**Affiliations:** Department of Urology, The First Affiliated Hospital, Zhejiang University School of Medicine, Hangzhou, China

**Keywords:** circRNAs, autophagy, regulation, cancer, progression

## Abstract

Circular RNAs (circRNAs) are a sort of long, non-coding RNA molecules with a covalently closed continuous ring structure without 5'-3' polarity and poly-A tail. The modulative role of circRNAs in malignant diseases has been elucidated by many studies in recent years *via* bioinformatics and high-throughput sequencing technologies. Generally, circRNA affects the proliferative, invasive, and migrative capacity of malignant cells *via* various mechanisms, exhibiting great potential as novel biomarkers in the diagnoses or treatments of malignancies. Meanwhile, autophagy preserves cellular homeostasis, serving as a vital molecular process in tumor progression. Mounting studies have demonstrated that autophagy can not only contribute to cancer cell survival but can also induce autophagic cell death in specific conditions. A growing number of research studies have indicated that there existed abundant associations between circRNAs and autophagy. Herein, we systemically reviewed and discussed recent studies on this topic in different malignancies and concluded that the circRNA–autophagy axis played crucial roles in the proliferation, metastasis, invasion, and drug or radiation resistance of different tumor cells.

## Introduction

Cancer is a global health problem that brings a huge burden to socioeconomic development. In 2021, approximately two million new cancer cases have been reported, with more than 600,000 predicted deaths in America alone (Siegel et al., 2021; Siegel et al., 2022). Great advancements have been achieved in the past several decades in the early diagnosis and treatment of cancers, leading to a remarkable decrease in overall incidence and mortality. However, in spite of more precise detective methods and progress in surgery, chemotherapy, and immunotherapy, the declining trend of cancer mortality started to stabilize in the late 1990s (Islami et al., 2021). A similar tendency occurs in China (Xia et al., 2022). As there has been no major breakthrough therapy providing a better prognosis for cancer in the past few years, novel mechanisms illustrating cancer etiology need urgently to be discovered for future developments of antineoplastic drugs or therapies.

In fact, circRNAs were regarded as waste RNAs generated from aberrantly spliced pre-mRNA when discovered in 1976 (Sanger et al., 1976; Liu et al., 2017). The gold mine of the biological characteristics of circRNAs was not tapped until 2013, when Hansen et al. (2013) discovered the sponging effect of ciRS-7 on miR-7 in mouse and human brains, starting the burgeoning time of circular RNA research.

**FIGURE 1:**
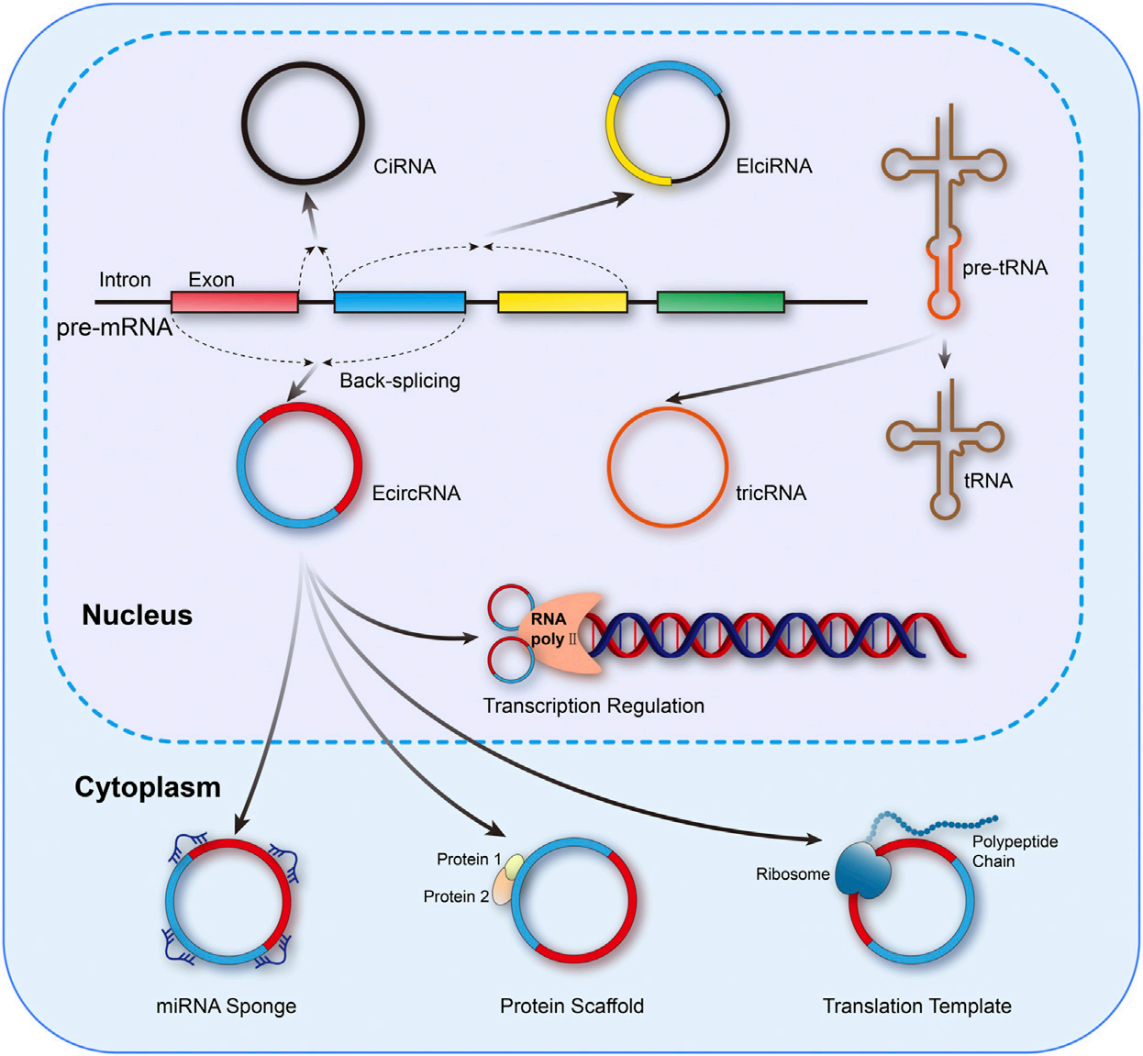
Mainstream formation and function mechanisms of circRNAs. circRNAs could be classified as EcircRNAs, EIciRNAs, CiRNAs, and tricRNAs. Meanwhile, the functional mechanisms of circRNAs mainly include miRNA sponges, binding to RNA binding proteins (RBPs), acting as protein scaffolds, regulation of transcription, and templates of translation for peptides.

The unique circular structure of circRNAs is generated from reverse splicing of the precursor mRNA (pre-mRNA), in which the 5’ or 3’ ends of the linear transcript are eliminated, endowing circRNAs with superior stability and resistance to RNase compared to linear counterparts (Zhong et al., 2018). According to the different formation mechanisms, circRNAs could be classified as exon circRNAs (EcircRNAs), exon–intron circRNAs (EIciRNAs), intron circRNAs (CiRNAs), and tRNA intronic circular RNAs (tricRNAs) (Zhou et al., 2020; van Zonneveld et al., 2021). In addition, circRNAs also exert crucial biological effects through varied mechanisms, including microRNAs (miRNAs) sponges, binding to RNA binding proteins (RBPs), acting as protein scaffolds, regulation of transcription, templates of translation for peptides, and even functioning as pseudogenes, which were exhibited in Figure 1 (Liu et al., 2017; Quan and Li, 2018; Zhong et al., 2018).

As a type of ubiquitous, conserved, and catabolic process, autophagy can be observed in almost all eukaryotic cells (Miller and Thorburn, 2021). After cells are stimulated by cellular stressors such as starvation or metabolic stress, AMPK is over-expressed, inducing the ULK1 complex subsequently. The ULK1 complex further phosphorylates Beclin 1 and upregulates its expression, leading to the formation of the PI3K VPS34 complex (Russell et al., 2013; Alizadeh et al., 2019). This is the initial step to constructing a phagophore. Mediated by lipidation of LC3-I, the emergence of autophagosome is completed, and then this machinery fuses with a lysosome, which is induced by LAMP2 and SNAREs (Paskeh et al., 2022). In autolysosomes, the pH-sensitive hydrolase degrades the intravesical cargos into small molecules and releases them into the cytoplasm for recycling of materials (Li et al., 2021a). In addition to the abovementioned signal pathway, other researchers confirm the inhibitory effect of the mTOR pathway in autophagy by suppressing the formation of phagophore membrane (Ashrafizadeh et al., 2022). In principle, autophagy could be classified as macroautophagy (herein referred to as autophagy), microautophagy, and chaperon-mediated autophagy (Miller and Thorburn, 2021; Xia et al., 2021).

**FIGURE 2:**
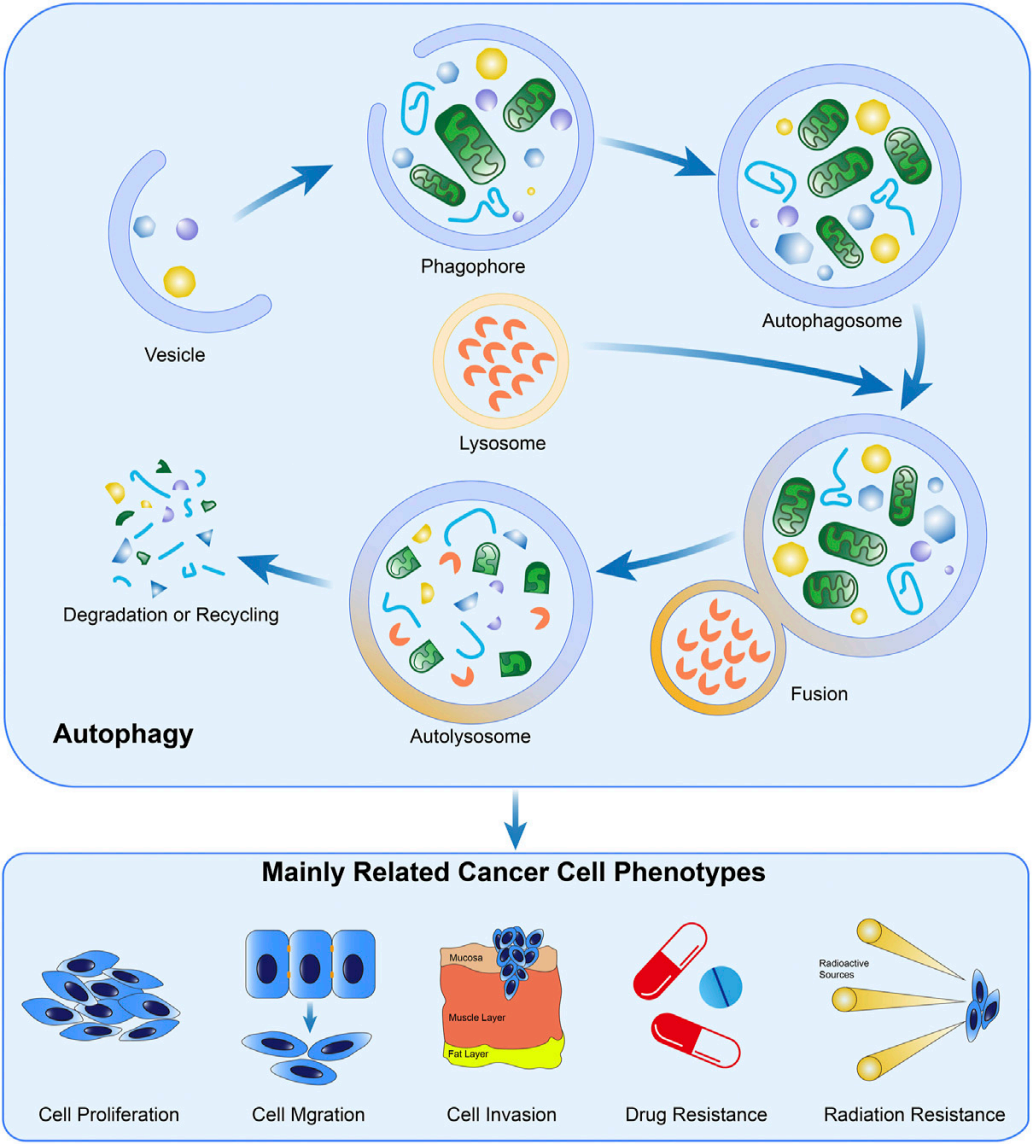
Formation and the biological functions of autophagy in cancer. The crucial steps in the formation of autophagy include autophagosome generation, the fusion of lysosomes and autophagosomes, and material degradation in autolysosomes. Evidence demonstrated autophagy is widely involved in the biological processes of cancer cells.

Physiologically, autophagy proceeds at a baseline level for the retainment of the intracellular homeostatic status and can be activated in the presence of various stressors (Kitada and Koya, 2021). Specifically, autophagy commensurately responds to oxidative stress, nutrient scarcity, and chemical toxicity, through efficiently mobilizing the intracellular energy and materials stored, benefiting cell survival (Yamazaki et al., 2021). However, accumulating evidence unveils the Janus-faced roles of autophagy in cellular biology, particularly in malignant diseases (Ishaq et al., 2020). Once autophagy is strengthened to an extent beyond the physiological threshold, the intracellular materials will be exhausted and the cell death program will, therefore, be initiated (Braicu et al., 2020), exerting the cancer-suppressive effects. The formation and biological functions of autophagy in cancer cells were presented in Figure 2.

Both circRNAs and autophagy widely participate in various biological activities involving promiscuous signaling pathways. Mounting explorations have proved that there exist potential associations between these two crucial regulative mechanisms (Zheng et al., 2020; Zhang et al., 2021a; Yu et al., 2021). Herein, we focus on the modulative effects of circRNAs which were mediated by the autophagy process in order to comprehensively explore the properties of circRNAs and autophagy as potential targets for cancer diagnosis and therapy.

## Main text

### CircRNA-autophagy network in cancers

**FIGURE 3:**
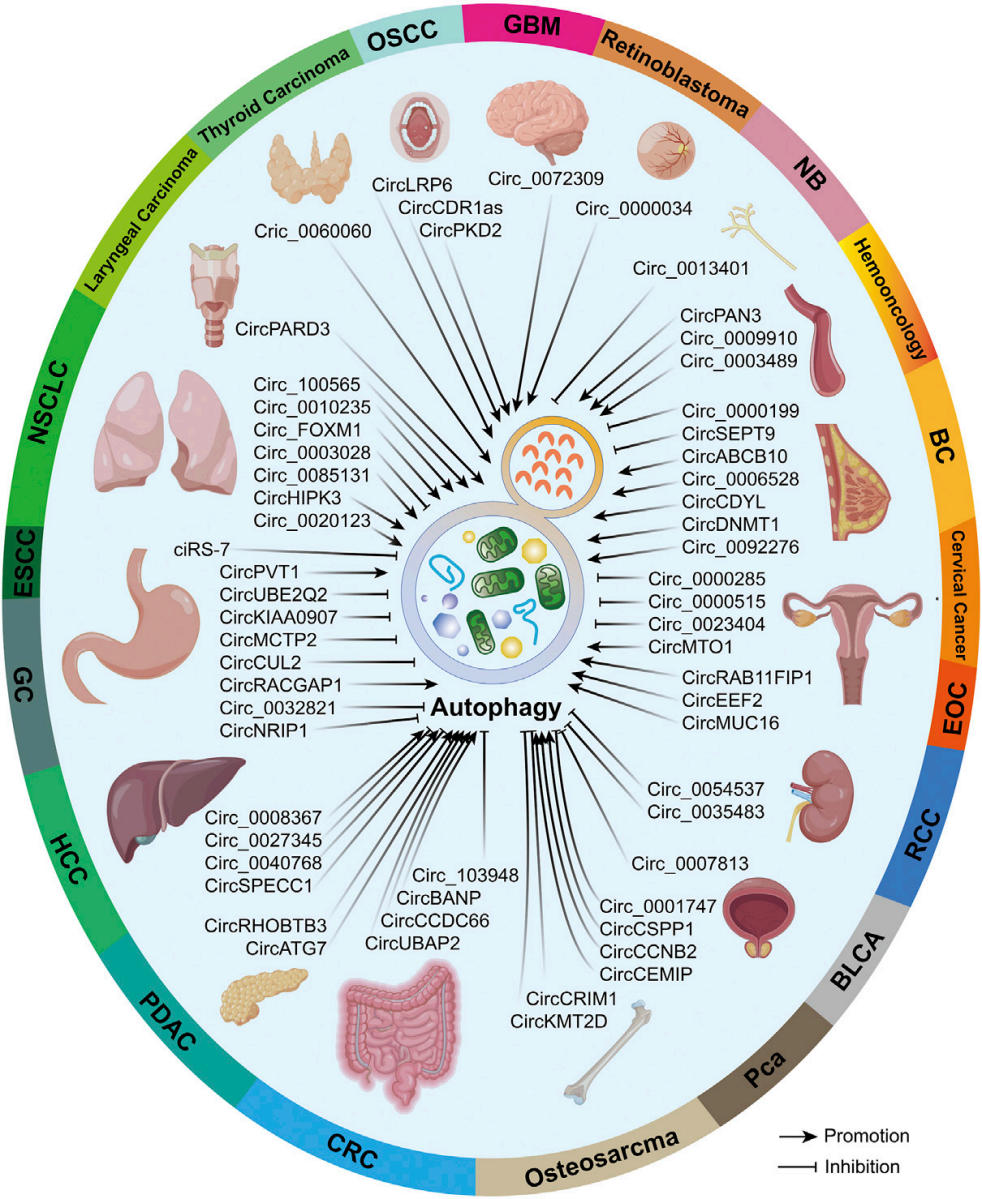
In different malignancies, circRNAs that can regulate autophagy were summarized. Partial materials were obtained from the online drawing tool (https://www.figdraw.com), with an authorization code IDTYUTSc5044.

Previous evidence proved that both circRNAs and autophagy possess high conservation and whether the circRNA–autophagy network plays pro- or anti-neoplasm effects is dependent on cancer type, pathological grade, clinical stage, and even exogenous stimuli (Kristensen et al., 2019; Zhou et al., 2021a). Hence, we believe it is essential to illustrate the circRNA–autophagy regulative system in specific malignancies, which was overviewed in Figure 3.

### Non-small cell lung cancer

Globally, lung cancer is the leading cause of all cancer-related deaths (Siegel et al., 2021; Sung et al., 2021), with non-small cell lung cancer (NSCLC) accounting for more than 85% of the newly diagnosed lung cancer cases (Herbst et al., 2018). Meanwhile, both circRNAs and autophagy are regarded as important modulators for the progression of NSCLC. Recent evidence found that circ_0010235, circ-FOXM1, circ_0085131, circ_0003028, circ_100565, circHIPK3, and circ_0020123 were all identified with high expressions in NSCLC cell lines or clinical samples and play tumor-genetic roles (Chen et al., 2020; Kong, 2020; Zhong et al., 2021a; Zhang et al., 2021b; Guan et al., 2021; Wei et al., 2021). Sponging effect is the mainstream regulating mechanism. Specifically, circ_0010235 sponged miR-433-3p to regulate the TIPRL levels, promoting the proliferation, autophagy, and migration of NSCLC (Zhang et al., 2021b); circ-FOXM1 and circ_0020123 exerted similar effects through the miR-149-5p/ATG5 and miR-512-3p/CORO1C axis, respectively (Wei et al., 2021; Zhang et al., 2022). In addition, cisplatin (DDP)-based chemotherapy has long been performed as an important adjuvant treatment for lung cancer, while DDP resistance in NSCLC brings great challenges to its curative effects. Silence of circ_0085131 was reported to inhibit the autophagy process through the miR‐654‐5p/ATG7 axis (Kong, 2020), which resulted in the increase in the sensitivity of NSCLC to DDP. Knockdown of circ_100565 can also restrain autophagy to mitigate the DDP resistance through sponging the miR-377-3p to upregulate the ADAM28 level, therefore providing a novel approach to deal with DDP resistance (Zhong et al., 2021a).

Notably, although circ_0003028 was proved to facilitate the proliferation, metastasis, and angiogenesis of NSCLC *via* modulation of the miR-1298-5p/GOT2 axis, the silence of circ_0003028 exhibited a promoting effect on autophagy, implying that circ_0003028 played an inhibitive role in autophagy (Guan et al., 2021). Intriguingly, circHIPK3 was exhibited to sponge miR-124-3p to regulate the downstream STAT3-PRKAA/AMPKα pathway, exerting a suppressive effect on autophagy in the A549 and H838 cell lines (STK11 mutant cell lines), while the facilitating role of circHIPK3 on autophagy was observed in H1299 cells (STK11 wild-type cell line), which was mediated through the promotive effect of circHIPK3 on STK11 (Chen et al., 2020).

### Esophageal squamous cell carcinoma (ESCC)

Pathologically, esophageal carcinoma consists of ESCC and esophageal adenocarcinoma (EAC), with the former accounting for nearly 88% of esophageal carcinoma cases (Murphy et al., 2017; Abnet et al., 2018). Large amounts of ncRNAs have been found to play crucial roles in the modulation of ESCC development (Feng et al., 2019). For example, Meng et al. (2020) proved that ciRS-7 was over-expressed in ESCC cell lines and tissues, playing an inhibitive role in rapamycin and starvation-induced autophagy. Furthermore, miR-1299 was shown to be sponged by ciRS-7 and exerted the effect of interfering with EGFR mRNA expression level by combining to the 3’UTR region of the EGFR mRNA followed by the EGFR-Akt-mTOR pathway effectively regulated, as well as the downstream autophagy process.

### Gastric cancer (GC)

Despite the great advances in treatment, patients suffering from GC mostly have an undesirable overall survival time, partly resulting from the late stage of GC at diagnosis (Peng et al., 2020). Both ncRNAs and autophagy were found to be the crucial regulators in the progression of GC (Wei et al., 2020; Xu et al., 2020; Rahman et al., 2021).

Over-expressed circKIAA0907 inhibited the proliferation, cell cycle, and cell autophagy in GC. Dual-luciferase report assays presented that circKIAA0907 sponged miR-452-5p, which negatively modulated the downstream KTA6B protein, weakening the repressive effects of circKIAA0907 in GC (Zhu et al., 2020). Moreover, circ_0032821 was proved to be highly expressed in GC tissues and cells, significantly associated with high clinical stages and poor prognostic results. Silence of circ_0032821 restrained the proliferation, EMT process, migration, and invasion, while enhancing the autophagy of GC cells. On the contrary, when circ_0032821 was over-expressed, the abovementioned phenotypes showed opposite trends, further confirming the oncogenetic roles of circ_0032821. The following explorations demonstrated that circ_0032821 activated the MEK1/ERK1/2 pathway to exert biological effects, while the specific mechanism of the activation of this axis was not discussed (Jiang et al., 2020).

Moreover, there have been large amounts of research studies demonstrating the roles of the circRNA–autophagy network in drug-resistant GC cells. For instance, Sun et al. (2020) found that circMCTP2 was notably repressed in the cisplatin (DDP)-resistant GC cells, and a high level of circMCTP2 predicted pleasant prognostic results. Moreover, the over-expressed circMCTP2 was proved to inhibit proliferation and autophagy while enhancing the sensitivity of GC cells to DDP. Further experiments proved that circMCTP2 bound miR-99a-5p through a competing endogenous RNA (ceRNA) mechanism to regulate the MTMR3 protein and exerted the abovementioned effects. Peng et al. (2020) also identified a low-expressed circCUL2 in GC tissue and cells, which was proved to repress the viability, autophagy, and metastasis of GC cells. Following experiments demonstrated that circCUL2 combined with miR-142-3p to modulate ROCK2 levels to interfere in the autophagy process, finally reducing the DDP resistance of GC cells.

Additionally, apatinib, a small molecular antiangiogenetic targeted agent, was proven to be safe and effective for GC patients when combined with oxaliplatin (Lin et al., 2021). Ma et al. (2020) presented that apatinib increased the levels of circRACGAP1 in GC cells, which sponged miR-3657 to regulate ATG expression and further induced autophagy to enhance GC cell resistance to apatinib. Therefore, silencing circRACGAP1 and inhibiting autophagy hold great potential for reinforcing the efficacy of apatinib in GC.

Almost all the cells could secret exosomes (Davis, 2016), and the circRNAs carried by exosomes were recognized as crucial regulators of distant malignant cells (Zhou et al., 2021b; Li et al., 2022). Yao et al. (2021) collected the serum of DDP-resistant GC patients and the culture medium of DDP-resistant GC cells, then identified the highly expressed circPVT1 in the exosomes of the abovementioned two systems. Knockdown of circPVT1 significantly refrained the autophagy and viability and increased the sensitivity of GC cells to DDP. Following rescue experiments proved that circPVT1 activates cell autophagy by regulating the miR-30a-5p/YAP1 axis. Similarly, Yang et al. (2021a) illustrated that circUBE2Q2 was highly expressed in GC cells and clinical samples and could be transported elsewhere by exosomes. Repression of circUBE2Q2 refrained the proliferation, glycolysis, invasion, and migration, while activating autophagy in GC cells. Further studies indicated that circUBE2Q2 sponged miR-370-3p to regulate the level of STAT3, exerting the abovementioned biological effects. Moreover, Zhang et al. (2019) found the upregulated circNRIP1 through the next generation sequencing of GC tissues and proved the promotive effect of circNRIP1 on the development of GC. miR-149-5p was regulated by circNRIP1 through a ceRNA mechanism and modulated the AKT1 level to influence the autophagy process. Exosomes carrying circNRIP1 were also proved to enhance the EMT and metastasis of GC cells in animal models.

### Breast cancer (BC)

In the female population, the number of annual new BC cases has surpassed that of lung cancer, becoming the most common malignancy (Sung et al., 2021). In particular, triple negative BC (TNBC) accounts for nearly 15% of all BC cases and is recognized as the most lethal BC due to its fast progression, high metastasis, and recurrence rate (Almansour, 2022; Derakhshan and Reis-Filho, 2022).

Zheng et al. (2020) identified the highly expressed circSEPT9 in TNBC cells and tissues, which positively correlated to the high clinical stages and poorer prognostic outcomes. Silencing of circSEPT9 played a repressive role in the proliferation and metastasis of TNBC cells, while activating autophagy. Subsequent experiments demonstrated that circSEPT9 sponged miR-637 to regulate the downstream target LIF/Stat3 signal pathway to exert the abovementioned effects. In addition, Liang et al. (2020) found high autophagy levels positively correlated to poorer clinical prognosis outcomes and identified the autophagy-related circCDYL. Over-expressed circCDYL was demonstrated to promote the autophagy process as well as the malignant biological behaviors, which were proved to be mediated *via* the downstream miR-1275-ATG7/ULK1 axis.

TNBC was found not sensitive to some anthracycline chemotherapy medicines (Chen et al., 2014), in which the circRNA–autophagy axis may play essential roles. For example, Li et al. (2021b) confirmed the existence of circAKT3, which was highly regulated in TNBC cells and tissues, positively related to increased tumor size, high clinical stages, and poorer prognostic outcomes. Over-expressed circAKT3 was proved to enhance the resistance of TNBC to DDP, ADM, PTX, and GEM. Following experiments demonstrated that circAKT3 could bind to miR-206 and miR-613 to interfere with their inhibition of the targeted PI3K/Akt/mTOR pathway, thus inhibiting the TNBC cell autophagy (Li et al., 2021b). Yang et al. (2020) attempted to explore the roles of highly regulated circABCB10 in BC cells (Liang et al., 2017) and demonstrated that silencing of circABCB10 markedly inhibited the resistance of BC cells to PTX as well as cell viability, invasion, and autophagy. Subsequent experiments suggested circABCB10 combined let-7a-5p to modulate downstream DUSP7 and further exerted the abovementioned effects (Yang et al., 2020). Based on previous research (Gao et al., 2019a), Liu et al. (2020a) attempted to investigate the roles of circ_0006528 in PTX-resistant BC cells. Results demonstrated that circ_0006528 was highly regulated in PTX-resistant BC cell lines. Inhibition of circ_0006528 notably suppressed the viability, metastasis, and cell autophagy of PTX-resistant BC cells. The following results suggested that miR-1299 could be combined with circ_0006528 to attenuate the inhibition of the CDK8 expression, thereby mediating the abovementioned roles of circ_0006528. Wang et al. (2021a) confirmed that upregulated circ_0092276 promoted ADM resistance by activating the autophagy process in BC cells. The functioning mechanism was sponging miR-348 to upregulate ATG7, which is one of the key genes in the autophagic pathway. Following experiments demonstrated that activated autophagy greatly contributed to its ADM resistance.

circRNAs could also directly bind to specific proteins to regulate their functions in BC (Salzman et al., 2012). For instance, circDNMT1 was upregulated in BC cell lines and clinical samples, with over-expression of circDNMT1, proved to enhance autophagy-mediated proliferation and viability. Meanwhile, circDNMT1 had the potency to combine with p53 protein to promote the relocation of p53 into cell nuclei, leading to the enhancement of cell autophagy and then subsequently enhanced cell viability. On the other hand, circDNMT1 could bind with AUF1 protein and promote the latter transported into cell nuclei, resulting in the inhibition of p53 expression. As p53 is an essential cancer suppressor (Du et al., 2018), circDNMT1 could also exert promotive effects on BC through the abovementioned mechanisms.

### Hepatocellular carcinoma (HCC)

Globally, primary hepatic carcinoma is the sixth most common and the third most deadly malignancy (Siegel et al., 2021; Sung et al., 2021). Pathologically, HCC accounts for 75–85% of all hepatic carcinoma cases (Janevska et al., 2015; Benson et al., 2021).

Sorafenib, a novel medicine with multiple targets, was widely applied for the treatment of late renal and liver cancers (Zhu et al., 2011). Liu et al. (2020b) identified a highly regulated circ_0008367 in sorafenib-treated HCC cells and demonstrated that circ_0008367 enhances cell autophagy and ferroptosis to mitigate the resistance of HCC cells to sorafenib. Further investigations suggested circ_008367 played the abovementioned roles through directly binding to ALKBH5 protein (Guo et al., 2018; Liu et al., 2020b), which was recognized as an autophagy inhibitor (Zhu et al., 2019). Matrine is a type of herbal compound with various biological effects, including suppressing the development of glioma and melanoma (Zhang et al., 2018; Zhou et al., 2018). Based on previous research (Lin et al., 2020), Lin et al. (2020) found that matrine markedly lowered the levels of circ_0027345, inducing cell apoptosis and autophagy in HCC cells. Furthermore, over-expressed circ_0027345 could regulate the miR-345-3p/HOXD3 pathway to inhibit the autophagy process, resulting in attenuation of the anti-tumor effect of matrine in HCC cells. In addition, circRNAs also mediate the effects of chemical compounds in the living cells. For example, Hao et al. (2020) proved that cadmium chloride upregulated the level of circ_0040768, which enhanced cell autophagy. Also, phenylethyl caffeate was proved to suppress the level of circ_0040768 and thus interfering the cadmium chloride from exerting toxic effects, providing a potential direction for the treatment of chromium poisoning. Meanwhile, Zhang et al. (2020a) found that H_2_O_2_ induced the downregulation of circSPECC1 in HCC, which was proved to interact with miR-33a to inhibit downstream autophagy, further promoting the progression of HCC in an oxidative situation, while the mechanism of miR-33a regulating autophagy was uninvestigated in this research.

### Prostate cancer (PCA)

In the global male population, PCA is the second most common and fifth most lethal cancer, depriving the lives of more than 370,000 patients every year (Mottet et al., 2021; Sung et al., 2021). The group of Chuanfang Zhong identified the autophagy-related circ_0001747 and proved that high levels of circ_0001747 positively correlated with a lower risk of biological recurrence of PCA. Silencing of circ_0001747 could activate the viability and autophagy of PCA cells (Zhong et al., 2021b). Lu et al. (2021) identified the upregulated circCSPP1 in PCA tissues and proved that high levels of circCSPP1 significantly enhanced the autophagy process as well as the proliferative and metastatic capability of PCA cells. miR-520h could be bound by circCSPP1 and affect the EGR1 expression to mediate the abovementioned effects. Yu et al. (2022) identified highly expressed circCEMIP in PCA cells and tissues, which functioned as a miR-1248 sponge to regulate downstream TM9SF4 protein expression level, further inhibiting mTOR phosphorylation and facilitating the autophagy process. Following experiments indicated that enhanced autophagy protected PCA cells from anoikis, accelerating PCA progression.

Radiotherapy plays an important role in the treatment of PCA, and the radical radiation therapy of PCA is markedly associated with the pleasant survival outcomes of PCA patients (Tharmalingam et al., 2019; Philippou et al., 2020). Through a review of previous research studies, Cai et al. (2022) learned that circCCNB2 could enhance the sensitivity of renal cell carcinoma to chemotherapy; therefore, they attempted to investigate whether circCCNB2 had similar effects in the radio-resistance of PCA. Following experiments demonstrated that circCCNB2 was upregulated in radio-resistant PCA cells, and silencing of circCCNB2 inhibited cell autophagy to increase the sensitivity of PCA cells to radiotherapy. miR-30b-5p could be combined with circCCNB2 and function as a regulator of KIF18A to mediate the abovementioned effects of circCCNB2 (Cai et al., 2022).

### Bladder cancer (BLCA)

Patients with BLCA usually suffer from a high rate of recurrence and poor clinical prognosis, especially for muscle-invasive bladder cancer (MIBC) (Flaig et al., 2018; Patel et al., 2020). The circRNA–autophagy network plays a crucial role in the progression of BLCA. Zhang et al. (2021c) identified upregulated circ_0007813 in BLCA tissues and proved that high expression of circ_0007813 significantly related to larger tumor size as well as higher clinical and pathological stages, predicting poorer clinical outcomes. Following experiments demonstrated that circ_0007813 was combined with miR-361-3p to regulate downstream IGF2R, further inhibiting the autophagy process of BLCA cells.

### Renal cell carcinoma (RCC)

Nearly 60–85% of renal carcinoma cases are pathologically diagnosed as RCC, among which 25% of cases are presented with distant metastasis at diagnosis (Chewcharat et al., 2019; Usher-Smith et al., 2020). Li et al. (2020a) learned about the abnormal expression of circ_0054537 in RCC and confirmed that repression of circ_0054537 observably suppressed the proliferation, metastasis, autophagy, and glycolysis of RCC cells, which was proved to be mediated by the miR-640/NPTX2 axis (Pei et al., 2021). Yan et al. (2019) identified upregulated circ_0035483 in RCC cells, which was proved to reinforce the autophagy of RCC cells through modulating the miR-335/CCNB1 axis. Moreover, the enhanced autophagy was shown to increase the viability of RCC in the administration of gemcitabine. Therefore, lowering the expression of circ_0035483 provides an inspiring strategy for combating gemcitabine resistance of RCC cells.

### Cervical cancer

Globally, cervical cancer has become the most common malignancy type in 23 regions and the most lethal cancer type in 36 regions (Sung et al., 2021). To further understand this disease, Tang et al. (2019), Guo et al. (2019), and Zhang and Zhang (2020) identified upregulated circ_0000515, circ_0023404, and circ_0000285 from cervical cancer tissue and cells, respectively. All the abovementioned circRNAs were proven to accelerate the growth and metastasis of cervical cancer, functioning through sponging miR-326 (targeting ELK1 mRNA), miR-5047, and miR-197-3p (targeting ELK1 mRNA), respectively. Notably, all these three circRNAs played an inhibitive role in the autophagy process. In addition, circ_0023404 also facilitated angiogenesis and DDP resistance in cervical cancer (Guo et al., 2019). Likewise, Jingjing Guo et al. also confirmed the highly expressed circMTO1, which presented effects of promoting the proliferative and metastatic potential of cervical cancer cells. Also, the miR-6893/S100A1 axis has been shown to mediate the aforementioned effects. In particular, circMTO1 activated the autophagy of cervical cancer cells and further enhanced the DDP resistance of cervical cancer cells (Chen et al., 2019).

### Colorectal cancer (CRC)

Annually, 10% of the newly diagnosed malignancy cases are proven to be colorectal cancer (Dekker et al., 2019), which ranks second in both the male and female population (Siegel et al., 2020).

The research group of Zhang et al. (2021d), Chen et al. (2021), and Xie et al. (2021), respectively, identified the upregulated circ_103948, circ_UBAP2, and circ_BANP in the CRC tissues and cells, and the following experiments proved that all the aforementioned circRNAs possessed the capability of promoting the colorectal cancer proliferation and metastasis. In particular, circBANP was shown to promote the radiation resistance of CRC. In terms of autophagy, circ_103948 presented obviously inhibitive effects, while the other two circRNAs were proved promotive. Subsequent experiments suggested circ_103948, circ_UBAP2, and circ_BANP could modulate miR-1236-3p/TPT1, miR-338-3p, and miR-582-5p/FOXO1 axis through the ceRNA mechanism, respectively, to exert the relevant biological effects.

Furthermore, Feng et al. (2020) also found that the hypoxic microenvironment could induce the high expression of circCCDC66, which enhanced autophagy and thus strengthened the proliferative and metastatic capability of CRC cells under a hypoxic environment.

### Pancreatic ductal adenocarcinoma (PDAC)

With the insidious onset, PDAC is usually found at the late stage of diagnosis, leading to a tumor that is unresectable through surgical method (Pant et al., 2019; Ho et al., 2020). Yang et al. (2021b) identified highly expressed circRHOBTB3 in PDAC cells and tissues, and their research suggested that circRHOBTB3 accelerated the viability and proliferation of PDAC cells, which was mediated by the circRHOBTB3-activated autophagy. Subsequent experiments demonstrated that circRHOBTB3 sponged miR-600 to upregulate the NACC1 expression, which further activated the Akt/mTOR pathway to induce cell autophagy. Meanwhile, He et al. (2022) discovered upregulation of circATG7 in PDAC tissue, which stabilized autophagy-related 7 (ATG7) mRNA by recruiting HUR and sponging miR-766-5p, a suppressor of ATG7 mRNA, thus increasing the expression of ATG7, which promotes the autophagy process.

### Ovarian cancer

Ovarian cancer is the most lethal gynecological malignancy (Hurwitz et al., 2021; Sung et al., 2021), and epithelial ovarian cancer (EOC) accounts for 95% of all ovarian cancer cases (Lheureux et al., 2019).

Gan et al. (2020) identified upregulated circEEF2 and circMUC16 from EOC tissues and cells, both of which promoted the proliferation and metastasis of EOC cells, as well as cell autophagy. Specifically, circEEF2 has been shown to combine with miR-6881-3p and modulate the downstream proteins, including ATG7 and ATG5, promoting autophagy course. Furthermore, circEEF2 was also proved to directly bind with ANXA2 protein, which could inhibit mTOR levels, resulting in enhanced autophagy (Yong et al., 2020). On the other hand, circMUC16 has been shown to sponge miR-199a, which could bind to mRNA of Beclin 1 and RUNX1 genes. CircMUC16 attenuated the inhibitive effect of miR-199a on Beclin 1 and RUNX1, exerting the promotive effect on autophagy. Further experiments also suggested that circMUC16 is directly bound to ATG13, contributing to enhanced autophagy.

Zhang et al. (2021a) identified upregulated circRAB11FIP1 from Torin1-pretreated EOC cells, which was proven to be highly expressed in EOC tissues (Lheureux et al., 2019). Silence of circRAB11FIP1 blocked the autophagic flux of EOC cells, while over-expressed circRAB11FIP1 exerted the promotive effect on autophagy and thus enhanced the proliferation and metastasis of EOC cells. Further experiments demonstrated that circRAB11FIP1 sponged miR-129 to upregulate ATG7 and ATG14, mediating the autophagy process. Moreover, circRAB11FIP1 has been shown to bind with FTO mRNA to enhance the FTO proteins, which could promote autophagy through methylation of m6A in ATG7 and ATG5 mRNAs.

### Neuroblastoma (NB)

With a high metastatic potential and malignant behaviors (Brodeur and Bagatell, 2014; Fletcher et al., 2018), NB presents poor prognostic outcomes, especially for junior patients of more than 18 months (DuBois and Park, 2018). Zhu et al. (2021a) identified high-regulated circ_0013401 in NB tissues and cells, the silence of which refrained the growth and metastasis of NB tissues but enhanced autophagy and apoptosis of NB cells. Further experiments demonstrated that circ_0013401 combined with miR-195 to regulate PAK2 expression.

### Retinoblastoma

RB is the most common intraocular tumor in infants and children (Broaddus et al., 2009). In RB cells, circ_0000034 downregulated miR-361-3p, a tumor suppressor, by a sponge mechanism (Liu et al., 2020c). Since the downstream gene of miR-361-3p, STX17, promoted the formation of autophagosomes, it is indicated that circ_0000034 accelerated the progress of RB by promoting the autophagy process.

### Glioma

Glioma is the most frequently occurring malignancy of the brain in adult patients, with glioblastoma (GBM) as the worst malignant pathological type (Tan et al., 2020; Yuan et al., 2022). Yuan et al. (2022) identified downregulated circ_0072309 in GBM and proved that over-expressed circ_0072309 upregulated RNF144B protein levels via sponging miR-100, leading to a decrease in ubiquitin-mediated p53 degradation, which enhanced autophagic cell death and TMZ sensitivity of GBM cells.

### Osteosarcoma

Osteosarcoma is the most common malignancy in the orthopedics department (Meltzer and Helman, 2021). Liu et al. (2020d) identified highly expressed circCRIM1 and proved that knockdown of circCRIM1 suppressed the growth and metastasis of osteosarcoma, activating autophagy at the same time. The miR-432-5p/HDAC4 axis has been shown to mediate the oncogenic roles of circCRIM1. Zhang et al. (2020b) found that H_2_O_2_ refrained the progression of osteosarcoma, while upregulating the circKMT2D in osteosarcoma. Following explorations demonstrated that silencing circKMT2D markedly impaired the viability and metastasis of osteosarcoma, while activating the autophagy process and strengthening the anti-cancer effect of H_2_O_2_.

### Hematological malignancies

Although chemotherapies and stem cell transplantation targeting malignant hematological diseases, including acute myeloid leukemia (AML), chronic myeloid leukemia (CML), and multiple myeloma (MM), have achieved significant progress in recent years, the prognosis of hematological cancers remains unsatisfactory (Carella et al., 2018; Szalat and Munshi, 2019; Stanchina et al., 2020).

In AML cells, researchers observed high expression of circ_0009910, knockdown of which could remarkably restrain their proliferation, sphere formation, and autophagy. Furthermore, evidence indicated that circ_0009910 directly bound miR-491-5p through a sponging mechanism, finally targeting gene B4GALT5 which alleviated the effects of upregulated miR-491-5p on cell proliferation (Wu et al., 2021). The same circ_0009910/miR-34a-5p sponge system also modulated ULK1-induced autophagy in imatinib-resistant CML cells (Cao et al., 2020). Another signaling axis was found in doxorubicin (ADM)-resistant AML cells. Over-expression of circPAN3 induced more autophagic activities through the AMPK/mTOR pathway, a well-known molecular signaling axis involved in autophagy (Altman et al., 2014; Shang et al., 2019), hence promoting the resistance of AML. With the silence of circPAN3, the autophagic activities were significantly downregulated and so was the drug resistance (Shang et al., 2019). These studies not only confirmed the close relationship between circRNAs and autophagy but also provided novel targets for drug-resistant leukemias. Moreover, downregulation of has_circ_0003489 was reported to alter the death pattern of MM cells *in vitro* from autophagy to apoptosis and inhibit their viability and cell proliferation ability through acting as a sponge of miR-874-3p, which targeted HDAC1. The circ_0003489/miR-874-3p/HDAC1 axis played an essential role in modulating the balance between autophagy and apoptosis in MM cells, providing a novel therapeutic strategy for MM (Tian et al., 2021).

### Head and neck cancers

The HNCs we discussed in this review included oral squamous cell carcinoma (OSCC) (Gao et al., 2019b; Cui et al., 2021a; Zhang et al., 2021e; Gao et al., 2022), laryngeal carcinoma (Gao et al., 2020), and thyroid carcinoma (Liu et al., 2018), in which circRNA–autophagy axis also played essential roles. Sponging-specific miRNAs are the mainstream regulative mechanisms, which could be found in Supplementary Table S1.

## Discussion

circRNAs have unique closed-looped and covalent structure without polarity ends, which endows the superior stability and resistance against RNases, making circRNAs potentially diagnostic or therapeutic targets in the treatment of cancers (Cui et al., 2018; Li et al., 2020b; van Zonneveld et al., 2021). Meanwhile, autophagy widely existed in almost all eukaryotes, playing essential roles in the material homeostasis in cancer cells (Chang et al., 2021; Kitada and Koya, 2021). A growing number of articles demonstrate the associations between these two processes, which may break a novel research ground (Zhou et al., 2021a; Wang et al., 2021b).

By far, although circRNAs could play biological roles in multiple ways, the mechanisms of circRNA regulating autophagy involve merely two types, namely, miRNA sponges and RBP binding. On the one hand, circRNAs combine with specific miRNAs activate crucial pathways to affect the autophagy process (Hansen et al., 2013; Zhong et al., 2018). For example, circEEF2 could bind with miR-6881-3p to upregulate ATG7 and ATG5 expression (Yong et al., 2020), promoting the autophagy process in ovarian cancer. On the other hand, circRNAs could directly bind with relative key proteins of autophagy initiation (Liu et al., 2017). For instance, circMUC16 was proven to directly bind with ATG13 to promote autophagy in ovarian cancer (Gan et al., 2020). Further exploration whether circRNAs could regulate the autophagy process through coding specific peptides and proteins or whether circRNAs could affect the expressions of crucial proteins in autophagy by regulating their transcription processes is required.

In most of the included articles, circRNAs promoted autophagy and exerted pro-cancer effects, facilitating the malignant biological activities, possibly through recycling intracellular proteins and organelles, conferring cancer cell survival advantages, and functioning as guardians of cancer cells (Miller and Thorburn, 2021). For example, highly expressed circCDYL promoted autophagy to promote the viability and proliferation of BC cells (Liang et al., 2020). However, autophagy is also regarded as one of the most complicated biological processes, exerting controversially paired effects in cancer biology (Cui et al., 2021b). In some conditions, excessive autophagy could also exhaust the intracellular materials and induce autophagic cell death, serving as an executioner of cancer cells (Kitada and Koya, 2021). Researchers also proved that autophagy could clean up the intracellularly toxic waste to protect the normal cells from tumor-genetic mutations, acting as a cancer suppressor (White et al., 2015; Li et al., 2020c). Those mechanisms could be the reason why some of the cancer-promoting circRNAs play suppressive roles in the regulation of autophagy. For instance, circPARD3 inhibited autophagy to accelerate the metastasis and invasion of laryngeal carcinoma, indicating that the anti-cancer roles of the circRNA–autophagy axis should not be ignored in the treatment of certain cancers (Gao et al., 2020).

The circRNA–autophagy axis also plays an essential role in the chemo- and radio-resistance of cancer cells. The frequent emergences of chemo- or radio-resistance of different cancers severely weaken the effects of these adjuvant therapies, during which process the roles of circRNAs regulated autophagy should be paid great attention. For instance, circPVT1 was proved to activate autophagy and increase the resistance of GC cells to DDP (Yao et al., 2021). Meanwhile, silencing of circCCNB2 was shown to significantly inhibit autophagy and further increase the radio-sensitivity of PCA cells (Cai et al., 2022). Under the abovementioned conditions and most of the malignancies, high autophagic activities are regarded as protective mechanisms in the chemo- or radio-resistant cancer cells, facilitating cancer survival in the chemo- or radio-treatment (Xia et al., 2021). Therefore, the combined administration of autophagy inhibitor with chemo- or radio-therapy will theoretically improve the clinical efficacy. Indeed, chloroquine, an autophagy inhibitor, was proven to mitigate the NSCLC cells’ resistance to erlotinib (Zou et al., 2013) and was co-administrated with radiation therapy for treating brain metastases, acquiring superior outcomes compared with radiation therapy alone (Rojas-Puentes et al., 2013). Hence, it is reasonable to hypothesize that targeting the circRNA–autophagy axis would greatly contribute to overcoming chemo- or radio-resistance of cancer cells (Ishaq et al., 2020; Mu et al., 2021).

In fact, with the development of pharmacology, a number of novel drugs have been identified for the treatment of malignancies, including targeted drugs and herbal medicines, such as apatinib, sorafenib, matrine, and so on (Liu et al., 2020b; Ma et al., 2020). The circRNA–autophagy axis has been shown to involve in the biological effects of the abovementioned medicines (Patra et al., 2020). For example, when administrated with apatinib, GC cells upregulated the expression of circRACGAP1 to enhance intracellular autophagy, obtaining survival advantages against apatinib treatment (Ma et al., 2020); matrine was proved to suppress the expression of circ_0027345 and further induce autophagy in HCC cells, inhibiting cellular proliferation and viability (Lin et al., 2020). All the aforementioned experiments demonstrate that targeting the circRNA–autophagy axis holds great potential in accelerating the clinical application of novel drugs.

Intriguingly, we demonstrated that the circRNA–autophagy axis involves various biological activities in cancer cells, including cell proliferation, migration, invasion, and drug and radio-resistance, and all the aforementioned biological processes are strongly related to cancer stem cells (CSCs) (Prasetyanti and Medema, 2017). CSCs are a small population of cancer cells that are identified in hematological malignancies and multiple solid tumors and characterized by the properties of self-renewability and pluripotency, giving rise to various cell phenotypes (Beck and Blanpain, 2013; Batlle and Clevers, 2017), which are responsible for anti-cancer therapy resistance, cancer recurrence, and metastasis (Nazio et al., 2019). Furthermore, both circRNA and autophagy were shown to actively participate in the regulation of CSCs. Autophagic homeostasis is reckoned indispensable for the maintenance of pluripotency of CSCs, serving as a crucial adaptative mechanism for CSCs under various stressors. For example, SOX2 was shown to increase the expression of Beclin 1 and facilitate the maturation of autophagosomes, promoting the EMT and CSC properties of colorectal cancer (Zhu et al., 2021b). Similarly, circRNAs also exert vital effects on the modulation of CSCs. For instance, circCPA4 was shown to upregulate PD-L1 expression and further enhance the stemness of NSCLC cells through sponging let-7 microRNA (Hong et al., 2020). Nevertheless, there are few research studies about whether circRNA could exert the regulative effects on CSCs through modulating autophagy, which severely hinders our understanding of deeper regulative mechanisms of cancer progression. Therefore, we appeal to more researchers to focus on this issue, which may potentially broaden our perspective on cancer progression.

Although there are no reported clinical trials for the use of circRNAs in the treatment of cancers at present, a bright perspective is expected by numerous researchers when it comes to the clinical application of the circRNA–autophagy axis for the treatment of various human diseases. Notably, circRNAs are enriched in cancer cells or extracellular exosomes, which makes them available as a treatment target or a novel diagnostic biomarker. For instance, a high level of hsa_circ_0095868 played oncogenic roles through regulating autophagy-related protein ATG16L1 and predicted poor clinical outcomes for HCC patients. Injection of β-amino esters encapsulated siRNA targeting hsa_circ_0095868 significantly reduced the volumes and lung metastasis of HCC in the mice model (Du et al., 2022). Meanwhile, circNRIP1 was found to motivate the GC cell survival and metastasis through regulating autophagy and was upregulated in the exosomes of GC patient plasma, making it a potential diagnostic or prognostic marker for the treatment of GC (Lu et al., 2020). Furthermore, based on the extraordinary stability of circRNAs, the latest study designed a circRNA vaccine against SARS-CoV-2 virus, which could highly express the trimeric receptor binding domain (RBD) of the spike protein of the virus, facilitating the human immune system to produce effective neutralizing antibodies against SARS-CoV-2 virus (Qu et al., 2022). This excellent work presented the possibility of developing novel circRNA vaccines against specific malignancy-specific proteins, further enhancing the immune reaction of the human body and restraining cancer development.

Despite the main bulk of the investigations focused on the regulative effects of circRNAs on autophagy, whether autophagy plays a role in the regulation of biological functions of circRNAs is less understood. Indeed, autophagy has been shown to participate in the degradation of specific kinds of nucleic acids, including ncRNAs and coding-RNAs, contributing to the homeostasis of nucleic acid balance. This uncanonical degradative mechanism was termed “RNautophagy” (Hase et al., 2015). For this novel type of autophagy, RNA was directly transported into lysosomes for further degradation in an ATP-dependent manner (Fujiwara et al., 2013), during which two membrane proteins of lysosomes played crucial roles, namely, LAMP2C and SIDT2 (Fujiwara et al., 2013; Hase et al., 2020). The cytosolic domain of both proteins possessed specific motifs, serving as RNA receptors and mediating the endocytosis of bound RNA into lysosomes, which is vital for the cytosolic RNA homeostasis. Notably, SIDT2 silencing was demonstrated to impair nearly half of the total degradative capacity for cytosolic RNA (Aizawa et al., 2016), implying the indispensable role of RNautophagy. In addition, researchers also found that autophagy receptor NDP52 facilitated the degradation of DICER and AGO2 proteins, both of which exerted crucial roles in the homeostasis of miRNAs. Repression of autophagy inhibited the degradation of DICER and AGO2 proteins, leading to the destabilization of miR-16 and let-7a (Gibbings et al., 2012). It is worth noting that autophagy is one of the two crucial degradative mechanisms for intracellular proteins, along with the ubiquitin-proteasome system (Kitada and Koya, 2021; Miller and Thorburn, 2021). Meanwhile, the formation of circRNAs also involves large amounts of RBPs, transcriptional regulators, and other bioactive proteins (Meng et al., 2017; Kristensen et al., 2019; Li et al., 2020b), whose abundance may be regulated by cell autophagy. Therefore, it is reasonable to hypothesize that autophagy could affect the formation or expression of circRNAs by regulating key regulative proteins of circRNAs. However, there are no published articles focusing on the issue. Further investigation is needed to determine whether autophagy could participate in the regulation of circRNAs and whether there existed a bilateral regulative mechanism between autophagy and circRNAs.

## Conclusion

In this review, we systematically reviewed the roles of the circRNA–autophagy axis in 22 malignancies. We concluded that circRNAs regulated autophagy mainly through sponging miRNAs and in combination with RBPs in most of the presented research studies, and the circRNA–autophagy axis exhibited controversial paired effects. Moreover, we believe that the circRNA–autophagy axis possesses great potential for clinical application. First, because the circRNA–autophagy axis deeply involves the chemo- and radio-resistance of cancer cells, regulating this axis may greatly enhance the efficacy of chemotherapy or radiotherapy; second, considering the circRNA–autophagy axis widely participates in the antineoplastic activities of novelly developed medicines, combined therapy of targeting the circRNA–autophagy axis with the administration of these medicines may multiply therapeutic effects. Nevertheless, whether there exists a bilateral regulative mechanism between autophagy and circRNAs and whether CSCs have vital roles in the circRNA–autophagy axis deserve deeper investigations.

## Abbreviations

ADM, Adriamycin; BTZ, bortezomib; DDP, cisplatin; EMT, epithelial–mesenchymal transition; GEM, gemcitabine; NA, not available; PTX, paclitaxel; SF, sorafenib.
