# Supplementary material for: Circular RNA-regulated autophagy is involved in cancer progression
Source: Front Cell Dev Biol. 2022 Sep 14;10:961983. doi: 10.3389/fcell.2022.961983 (PMC9515439; doi:10.3389/fcell.2022.961983)
Supplement: Supplementary file 1 [file Table1.pdf]

Cancer Types	CircRNAs	Level	Downstream Targeted Pathways	Functioning Mechanism	Effects on Autophagy	Biological Effect of circRNAs on Cancer cells Phenotypes	Cell lines	References
NSCLC	Circ_100565	Up	miR-377-3p/ADAM28	miR Sponge	Promotion	Promote viability and DDP resistance	A549, H1299	(Zhong et al., 2021b)
	Circ_0010235	Up	miR-433-3p/TIPRL	miR Sponge	Promotion	Promote proliferation and migration. Inhibit apoptosis	A549, H1299	(Zhang et al., 2021a)
	Circ-FOXM1	Up	miR-149-5p/ATG5	miR Sponge	Promotion	Promote proliferation and migration. Inhibit apoptosis	A549, H1581	(Wei et al., 2021)
	Circ_0003028	Up	miR-1298-5p/GOT2	miR Sponge	Inhibition	Promote proliferation, migration invasion, and angiogenesis	A549, H1299	(Guan et al., 2021)
	Circ_0085131	Up	miR-654-5p/ATG7	miR Sponge	Promotion	Promote the DDP resistance	A549	(Kong, 2020)
	CircHIPK3	Up	miR-124-3p/ STAT3-PRKAA/AMPKα	miR Sponge	Inhibition	Promote proliferation, migration and invasion	A549, H838	(Chen et al., 2020)
			STK11	RBP[1]	Promotion		H1299	
	Circ_0020123	Up	miR-512-3p/CORO1C	miR Sponge	Promotion	Promote proliferation, motility and angiogenesis	A549, PC9	(Zhang et al., 2022)
ESCC	ciRS-7	Up	miR-1299/EGFR	miR Sponge	Inhibition	Promote viability	Eca109, TE1, KYSE170	(Meng et al., 2020)
GC	CircPVT1	Up	miR-30a-5p/YAP1	Exosome; miR-sponge	Promotion	Promote viability, invasion and DDP resistance	HGC27, AGS	(Yao et al., 2021)
	CircUBE2Q2	Up	miR-370-3p/STAT3	Exosome; miR-sponge	Inhibition	Promote proliferation, migration, invasion, EMT, and glycolysis	MKN45, BGC823 HGC27, AGS	(Yang et al., 2021a)
	CircKIAA0907	Down	miR-452-5p/KAT6B	miR-sponge	Inhibition	Inhibit viability, proliferation and cell cycle	BGC823,	(Zhu et al., 2020)
	CircMCTP2	Down	miR-99a-5p/MTMR3	miR-sponge	Inhibition	Inhibit viability, proliferation, and DDP resistance	SGC7901	(Sun et al., 2020)
	CircCUL2	Down	miR-142-3p/ROCK2	miR-sponge	Inhibition	Inhibit viability, proliferation, migration and invasion	SGC7901, AGS	(Peng et al., 2020)
	CircRACGAP1[1]	Up[1]	miR-3657/ATG7	miR-sponge	Promotion	Inhibit apoptosis, Apatinib resistance	BGC823, HGC27	(Ma et al., 2020)
	Circ_0032821	Up	MEK1/ERK1/2	NA	Inhibition	Promote proliferation, EMT, migration and invasion	AGS, HGC27	(Jiang et al., 2020)
	CircNRIP1	Up	miR-149-5p/AKT1/ mTOR	Exosome, miR-sponge	Inhibition	Promote proliferation, migration and invasion	MKN45, BGC823	(Zhang et al., 2019)
BC	Circ_0000199	Up	miR-206/613-led PI3K/Akt/mTOR	miR-sponge	Inhibition	Promote proliferation, migration invasion and chemo-resistance (DDP, ADM, PTX and GEM)	MDA-MB-231, MDA-MB-468	(Li et al., 2021a)
	CircSEPT9	Up	miR-637/LIF/STAT3	miR-sponge	Inhibition	Promote proliferation, migration and invasion	MDA-MB-231, BT-549	(Zheng et al., 2020)
	CircABCB10	Up	let-7a-5p/DUSP7	miR-sponge	Promotion	Promote viability, invasion and PTX resistance	MDA-MB-231, MCF-7	(Xu et al., 2020b)
	Circ_0006528	Up	miR-1299/CDK8	miR-sponge	Promotion	Promote viability, proliferation, migration invasion and PTX resistance	BT-549, ZR-75	(Liu et al., 2020a)

	CircCDYL	Up	miR-1275-ATG7/ULK1	miR-sponge	Promotion	Promote viability and proliferation	MDA-MB-231, MCF-7	(Liang et al., 2020)
	CircDNMT1	Up	p53 nuclear translocation	RBP	Promotion	Promote viability and proliferation	MCF-7	(Du et al., 2018)
	Circ_0092276	Up	miR-348/ATG7	miR-sponge	Promotion	Promote viability and proliferation	MCF-7, MDA-MB-468	(Wang et al., 2021a)
HCC	Circ_0008367[2]	Up[2]	ALKBH5	RBP	Promotion	Promote ferroptosis	HepG2, Huh7	(Liu et al., 2020d)
	Circ_0027345[3]	Down[3]	miR-345-5p/HOXD3	miR-sponge	Inhibition	Promote the viability and proliferation	Huh7, HCCLM3	(Lin et al., 2020)
	Circ_0040768[4]	Up[4]	NA	NA	Promotion	Inhibit viability and proliferation	HepG2	(Hao et al., 2020)
	CircSPECC1[5]	Down[5]	miR-33a	miR-sponge	Inhibition	Promote viability and proliferation	HepG2, Huh7	(Zhang et al., 2020a)
PCA	Circ_0001747	Down	NA	miR-sponge	Inhibition	Inhibit viability and proliferation	22RV1, DU145	(Zhong et al., 2021a)
	CircCSPP1	Up	miR-520h-EGR1	miR-sponge	Promotion	Promote viability, proliferation, migration invasion	PC3, DU145	(Lu et al., 2021)
	Circ_CCNB2	Up	miR-30b-5p/KIF18A	miR-sponge	Promotion	Promote viability, proliferation, migration, invasion and radio-therapy resistance	DU145, LNCap	(Cai et al., 2020)
	CircCEMIP	Up	miR-1248/TM9SF4	miR-sponge	Promotion	Promote viability, proliferation, migration, invasion; Protect PCA from anoikis	PC-3, DU-145	(Yu et al., 2022)
BLCA	Circ_0007813	Up	miR-361-3p/IGF2R	miR-sponge	Inhibition	Promote proliferation, migration and invasion	T24, UM-UC-3	(Liu et al., 2021)
RCC	Circ_0054537	Up	miR-640/NPTX2	miR-sponge	Promotion	Promote proliferation, migration, invasion, and glycolysis	786O, A498	(Pei et al., 2021)
	Circ_0035483	Up	miR-335/CCNB1	miR-sponge	Promotion	Promote the viability and GEM resistance	TK10, UO31	(Yan et al., 2019)
Cervical cancer	Circ_0000285	Up	miR-197-3p/ELK1	miR-sponge	Inhibition	Promote viability, proliferation	SiHa, Hela	(Zhang and Zhang, 2020)
	Circ_0000515	Up	miR-326/ELK1	miR-sponge	Inhibition	Promote viability and invasion	SiHa, Hela	(Tang et al., 2019)
	Circ_0023404	Up	miR-5047	miR-sponge	Inhibition	Promote viability, invasion, DDP resistance and lymphatic vessel formation	SiHa, Hela	(Guo et al., 2019)
	CircMTO1	Up	miR-6893	miR-sponge	Promotion	Promote viability, invasion and DDP resistance	SiHa, Hela	(Chen et al., 2019)
CRC	Circ_103948	Up	miR-1236-3p/TPT1	miR-sponge	Inhibitioiin	Promote viability, proliferation, migration and invasion	HCT-116, SW480	(Zhang et al., 2021b)
	CircBANP	Up	miR-338-3p	miR-sponge	Promotion	Promote viability, proliferation and radio therapy resistance	LoVo	(Xie et al., 2021)
	CircCCDC66[6]	Up[6]	miR-3140	miR-sponge	Promotion	Promote viability, migration and invasion	HCT-116, SW620	(Feng et al., 2020)
	CircUBAP2	Up	miR-582-5p/FOXO1	miR-sponge	Promotion	Promote proliferation, migration and invasion	HCT-116, SW480	(Tang et al., 2021)
PDAC	CircRHOBTB3	Up	miR-600/NACC1	miR-sponge	Promotion	Promote the proliferation	PANC-1, MiaPaCa-2	(Yang et al., 2021b)
	CircATG7	Up	miR-766-5p/ATG7 HUR	miR-sponge RBP	Promotion	Promote viability, proliferation, migration and invasion	PANC-1, MiaPaCa-2	(He et al., 2022)

EOC	CircRAB11FIP1[7]<br><br><br><br>Circ-EEF2<br><br><br>CircMUC16	Up[7]<br><br><br><br>Up<br><br><br>Up	miR-129/ (ATG7 and ATG14)<br>FTO/ (ATG7 and ATG5) m6A<br>miR-6881-3p/ (ATG7 and ATG5)<br>ANXA2<br>miR-199a-5p/ (Beclin1 and RUNX1)<br>ATG13	miR-sponge<br><br>Binding to FTO mRNA<br><br>miR-sponge<br><br>RBP<br>miR-sponge<br><br>RBP	Promotion<br><br><br><br>Promotion<br><br><br>Promotion	Promote proliferation and migration<br><br><br><br>Promote proliferation and invasion<br><br><br>Promote migration and invasion	A2780, SKOV3<br><br><br><br>A2780, SKOV3<br><br><br>A2780, SKOV3	(Zhang et al., 2021d)<br><br><br><br>(Yong et al., 2020)<br><br><br>(Gan et al., 2020)
NB	Circ_0013401	Up	miR-195/PAK2	miR-sponge	Inhibition	Promote proliferation, migration and invasion	SH-SY5Y, SK-N-BE	(Zhu et al., 2021a)
Osteosarcoma	CircKMT2D[8]<br>CircCRIM1	Up[8]<br>Up	miR-210<br>miR-432-5p/HDAC4	miR-sponge<br>miR-sponge	Inhitition<br>Inhibition	Promote viability and invasion<br>Promote proliferation, migration and invasion	MG63, U2OS<br>MG63, U2OS	(Zhang et al., 2020b)<br>(Liu et al., 2020c)
AML	Circ_0009910<br><br>CircPAN3	Up<br><br>Up	miR-491-5p/B4GALT5<br>miR-491-5p/PI3K/AKT<br>AMPK/mTOR	miR-sponge<br><br>NA	Promotion<br><br>Promotion	Promote proliferation, sphere formation<br><br>Promote ADM resistance	HL60, MOLM13<br><br>THP-1, K562	(Wu et al., 2021)<br><br>(Shang et al., 2019)
CML	Circ_0009910	Up	miR-34a-5p/ULK1	miR-sponge	Promotion	Promote proliferation and imatinib-resistance	K562	(Cao et al., 2020)
MM	Circ_0003489	Up	miR-874-3p/HDAC1	miR-sponge	Promotion	Promote viability, proliferation and BTZ resistance	MM1.R	(Tian et al., 2021)
Retinoblastoma	Circ_0000034	Up	miR-361-3p/STX17	miR-sponge	Promotion	Promote proliferation, migration and invasion	Y79, WER1-Rb1	(Liu et al., 2020b)
GBM	Circ_0072309	Down	miR-100/RNF144B	miR-sponge	Promotion	Inhibit viability and proliferation	U87, U251, A172	(Yuan et al., 2022)
OSCC	Circ-LRP6<br>CircCDR1as<br>CircCDR1as[9]<br><br>CircPKD2	Up<br>Up<br>Up[9]<br><br>Down	NA<br>miR- 876- 5p/SLC7A11<br>AKT/ERK½/mTOR<br>miR-671-5p<br>miR-646/ATG13	NA<br>miR-sponge<br>NA<br>miR-sponge<br>miR-sponge	Promotion<br>Promotion<br>Promotion<br><br>Promotion	Promote EMT, migration and invasion<br>Promote viability, proliferation and cell cycle<br>Promote viability, endoplasmic reticulum stress<br>Inhibit viability, proliferation, DDP resistance	SCC-15<br>SCC9, Cal-27<br>Tca-8113, SCC-15<br>SCC-15, Cal-27	(Zhang et al., 2021c)<br>(Cui et al., 2021a)<br>(Gao et al., 2019b)<br><br>(Gao et al., 2022)
Laryngeal carcinoma	CircPARD3	Up	miR-145-5p/PRKCI-Akt-mTOR	miR-sponge	Inhibition	Promote proliferation, migration, invasion and DDP chemoresistance	FD-LSC-1, Tu177	(Gao et al., 2020)
Thyroid carcinoma	Circ_0060060	Up	miR-144-3p/TGF-α	miR-sponge	Promotion	Promote proliferation and DDP resistance	TPC1, BHT101	(Liu et al., 2018)

Annotations: [1]Apatinib induced; [2]SF induced; [3]Matrine induced; [4]CdCl2 induced; [5]H_2O_2 induced, [6]Hypoxia induced; [7]Torin 1 induced; [8]H_2O_2 induced; [9]Hypoxia induced. Abbreviation: EMT: epithelial-mesenchymal transition; DDP: cis-platin; PTX: paclitaxel; SF: sorafenib; ADM: adriamycin; BTZ: bortezomib; GEM: gemcitabine; NA: not available.
